# Identification of PPT1 as a lysosomal core gene with prognostic value in hepatocellular carcinoma

**DOI:** 10.1042/BSR20230067

**Published:** 2023-05-18

**Authors:** Wei Tian, Chenyu Li, Jiaqi Ren, Pengfei Li, Jingyuan Zhao, Shuai Li, Deshi Dong

**Affiliations:** 1 The First Affiliated Hospital of Dalian Medical University, Dalian, China; 2 Dalian Medical University, Dalian, China; 3 Regenerative Medicine Center, The First Affiliated Hospital of Dalian Medical University, Dalian, China; 4 Department of Pharmacy, The First Affiliated Hospital of Dalian Medical University, Dalian, China

**Keywords:** bioinformatics, Hepatocellular carcinoma, lysosomal, PPT1, proteomics

## Abstract

Hepatocellular carcinoma (HCC) is the most frequent cancer worldwide with a poor prognosis. Unfortunately, there are few reports on effective biomarkers for HCC, identification of novel cancer targets is urgently needed. Lysosomes are central organelles for degradation and recycling processes in cells, and how lysosome-related genes are involved in the progression of hepatocellular carcinoma remains unclear. The aim of the present study was to identify key lysosome-related genes affecting HCC. In the present study, lysosome-related genes involved in HCC progression were screened based on the TCGA (The Cancer Genome Atlas) dataset. Differentially expressed genes (DEGs) were screened, and core lysosomal genes were obtained in combination with prognostic analysis and protein interaction networks. Two genes were associated with survival, and their prognostic value was validated by prognostic profiling. After mRNA expression validation and IHC, the palmitoyl protein thioesterase 1 (PPT1) gene was identified as an important lysosomal-related gene. We demonstrated that PPT1 promotes the proliferation of HCC cells *in vitro*. In addition, quantitative proteomics and bioinformatics analysis confirmed that PPT1 acts by affecting the metabolism, localization, and function of various macromolecular proteins. The present study reveals that PPT1 could be a promising therapeutic target for the treatment of HCC. These findings provided new insights into HCC and identified candidate gene prognosis signatures for HCC.

## Introduction

Hepatocellular carcinoma (HCC) is one of the most common malignant tumors of the digestive system with high malignancy and poor prognosis, and the fourth leading cause of cancer-related deaths [1]. The current treatment measures for hepatocellular carcinoma are mainly surgical, but the recurrence rate at 5 years after surgery is high and the survival rate is low. Despite new breakthroughs in interventional radiology, surgical techniques, and liver transplantation in recent years, the prognosis of advanced hepatocellular carcinoma is still very poor and there is no effective treatment [2,3]. Therefore, further clarification of the molecular mechanisms of hepatocellular carcinoma can help in early diagnosis, prognosis prediction, and precise tumor treatment.

Lysosomal plays a remarkable role in maintaining and regulating the homeostasis of intracellular material and energy metabolism due to its attractive ability to degrade multiple substrates [4,5]. The lysosomal degradation pathway is an important regulatory mechanism for cellular and intra-organismal homeostasis, mediating a variety of healthy cellular biological processes, as well as being involved in many complex tumor biological processes such as nutrient sensing, cell signaling, cell death, immune response, and cellular metabolism [6]. Lysosomal degradation is also an intracellular self-protection mechanism, especially triggered during nutrient or energy deficiency [7]. In addition, the cell can also defend against oxidative stress through the lysosomal degradation pathway [8,9]. Lysosomal can reduce cellular damage by removing potential toxic substances and increasing cell adaptability. The lysosome is a key organelle and cellular target in the autophagic process [10]. Studies have demonstrated that lysosome dysfunction is associated with the development of several diseases, such as neurodegenerative diseases and tumors [11–15]. Targeting lysosomes not only triggers lysosome-dependent cell death to kill cancer cells but also intervenes in cancer cell survival by regulating autophagy [16]. Therefore, the research on the mechanism of lysosome regulating disease development and the treatment targeting the lysosomal pathway is worthy of further exploration.

In the present study, bioinformatics analysis of TCGA data from HCC was performed to screen differentially expressed lysosomal genes for the construction of prognostic models and to analyze the effects of genes on pathways and tumor immunity. PPT1, as a lysosomal-related gene with prognostic value, was experimentally analyzed for its expression in HCC patients and its effects on tumor cell proliferation were investigated. The combination of quantitative proteomics and bioinformatics analysis is a good strategy to explore the molecular mechanisms of biology [17]. By performing quantitative proteomics and functional assays *in vitro*, the pathways affected by PPT1 inhibitors and the role and mechanisms by which they exert their anti-tumor effects were investigated.

## Materials and methods

### Data abstraction and differential gene expression analysis

The gene expression profiles of HCC were downloaded from TCGA. Lysosome-related genes (LRGs) were obtained from the Molecular Signatures Database (MSigDB). We used the limma package in R software to screen the differential genes (DEGs) between tumor samples and normal tissues. Only genes with an Index of |log 2 [FC]| > 1 and a corrected *P*<0.05 were identified as DEGs. On this basis, DEGs were carried out for further protein–protein interaction (PPI) network construction which was constructed by the STRING website and Cytoscape.

### Construction of the prognostic risk gene signature

The best prognosis LRGs were selected by LASSO analysis using R software. Using multivariate cox regression analysis to establish a risk model. The calculation formula of Risk score: Risk score (RS) = Σ gene expression × coefficient, according to the median, the calculated RS values are divided into two groups, namely the high-risk group and the low-risk group. To assess the sensitivity and specificity of the model, the ROC curves were drawn at 1, 3, and 5 years. Then calculate the area under the curve corresponding to three times, that is, the AUC value. At last, the OS of the various risk groups were shown by Kaplan–Meier survival curves to assess the performance of TFs-based signature.

### Enrichment analysis of DEGs between high-risk and low-risk groups

In order to identify DEGs, the limma package of R software was used to compare the expression profiles of the high-risk group and the low-risk group. Gene Ontology (GO) and Kyoto Encyclopedia of Genes and Genomes (KEGG) pathway enrichment analyses of the DEGs were performed using the ‘ClusterProfiler’ R package.

### Tumor-infiltrating immune cell profile

CIBERSORT determines the proportion and abundance of different types of immune cells in the mixed cell population based on the data of gene expression. To explore the degree of immune cell infiltration, CIBERSORT was used to evaluate the fractions of immune cell types in liver cancer samples and analyze the difference between high- and low-risk groups.

### Validation of the PPT1 in clinical tissue samples and HPA database

A total of 20 paired HCC patient specimens were collected from the Biobank of the First Affiliated Hospital of Dalian Medical University (Liaoning, China). The samples were removed from hospitalized patients at the Department of Hepatobiliary Surgery, First Affiliated Hospital of Dalian Medical University (Liaoning, China) from 2018 to 2021. Samples used in the present study were approved by the Committees for Ethical Review of Research at the First Affiliated Hospital of Dalian Medical University. The PPT1 gene mRNA expression between tumor and normal groups was assayed by real-time PCR.

The validation of the protein levels of the key genes was carried out using the Human Protein Atlas (HPA) database (https://www.proteinatlas.org/).

### RNA extraction and quantitative real-time PCR

According to the manufacturer’s manuals, we use the trizol reagent (Thermo Fisher) to extract RNA. The cDNA was synthesized by the RevertAid Master Mix Reagen. Real-time quantitative PCR analyses were processed via the SYBR Green PCR Master Mix (Thermo Fisher), and GAPDH was regarded as a control. The 2^−ΔΔCt^ method was used to compare the fold differences in expression. Primer sequences were listed as follows: PPT1 forward 5′-TGTTTTTGGACTCCCTCGATG-3′ and reverse 5′-CATGCCAGTATTCGGCTTGC-3′, GAPDH forward 5′- ACAACTTTGGTATCGTGGAAGG-3′ and reverse 5′- GCCATCACGCCACAGTTTC-3′.

### Cell culture

Human HCC cell lines MHCC-97H and HuH-7 were obtained from the Chinese Type Culture Collection, Chinese Academy of Sciences. All cells were grown in Dulbecco’s Modified Eagle Medium (DMEM) medium supplemented containing 10% fetal bovine serum (FBS) and 1% Penicillin–Streptomycin, which was maintained at 37 °C in a humidified air with 5% of CO_2_.

### Cell proliferation assays

The dissolved DC661 (MedChemExpress, U.S.A., dissolves DC661 with DMSO to 1 mM) was diluted to a working concentration with complete medium. Cells in the logarithmic growth phase were inoculated into 96-well plates and treated with DC661 for 24 h. Three replicate wells were set up for each group, and cell proliferation was determined using Cell Counting Kit 8 (CCK8, APExBIO, U.S.A.), according to the manufacturer’s instructions.

### Colony formation

500 HCC cells were inoculated into each well of a 6-well plate and maintained in a medium containing 10% FBS for 10 days. Colonies were fixed with 4% paraformaldehyde, stained with 0.1% crystal violet solution, and counted under an inverted microscope. Three replicate wells were set up for each group of experiments.

### Western blot

To prepare total protein, the lysate was prepared with RIPA and PMSF (Beyotime, China) in a ratio of 100:1 on ice. The mixed solution was added to the Petri dish cleaned with ice-cold PBS, and the cells were lysed in the lysate on ice for 30 min. After centrifugation at 4°C, the supernatant was obtained and the concentration of protein samples was determined. The protein samples were boiled and frozen. A 12% sodium dodecyl sulfate polyacrylamide gel was prepared, then the same amount of protein samples were injected into the gel and were electro-transferred onto the polyvinylidene difluoride (PVDF) membranes (Millipore, U.S.A.). When membrane transformation was finished, PVDF membranes were blocked with a 4% blocking solution prepared by TBST and albumin bovine V (Solarbio, China), primary antibodies were incubated overnight at 4°C: anti-LC3B (1:1000, ABclonal, China), anti-ATG5 (1:1000, ABclonal, China) and anti-β-actin (1:1000, ABclonal, China), and secondary antibodies were incubated at room temperature.

### Mass spectrometry and bioinformatics analyses

Mass spectrometry and bioinformatics analyses Mass spectrometry was performed by Novogene (Beijing, China). The threshold set for up-and down-regulated protein was a fold change ≥ ×2.0 and a *P* <0.05. MHCC-97H cells were treated with 2 mM DC661 for 48 h, and the cell lysates were homogenized in RIPA lysis buffer.

### Statistical analyses

GraphPad Prism 7 software was used for statistical analysis. The statistical analysis involved in the reported data met the criteria of using appropriate statistical tests. The one-way ANOVA or Student’s *t*-test was used for analysis, and *P*<0.05 was considered statistically different.

## Result

### Identification of differentially expressed LRGs in HCC

**Figure 1:**
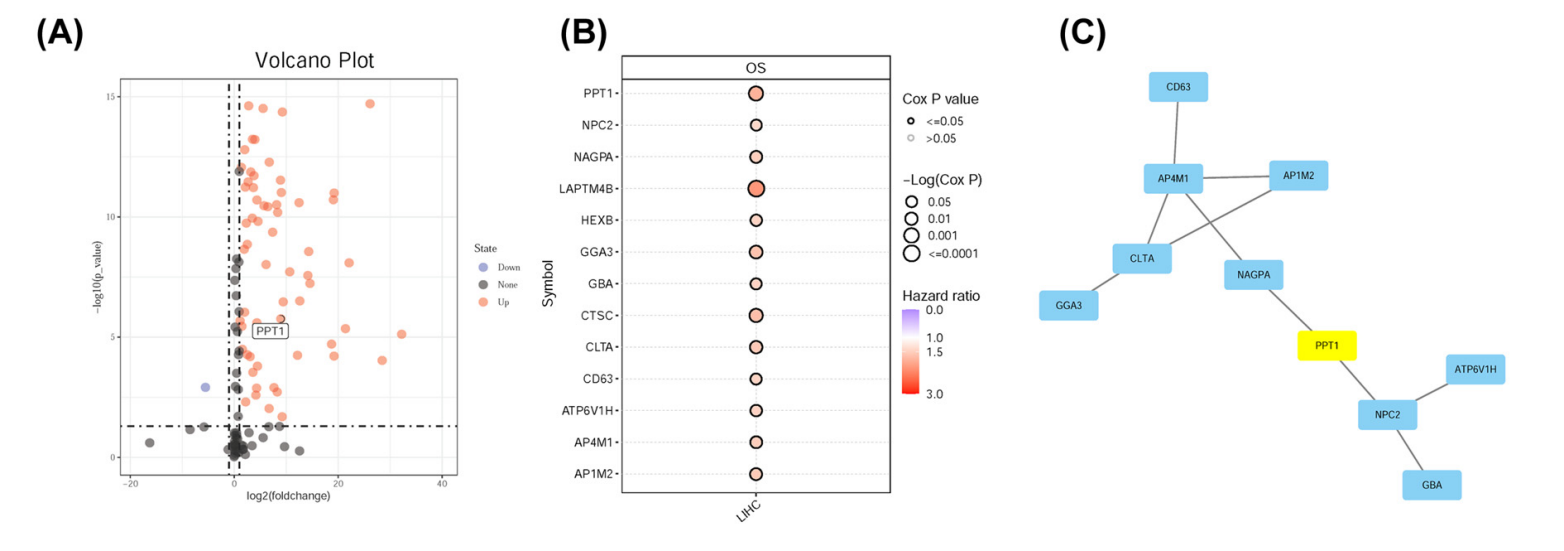
Identification of DEGs between tumor samples and normal tissues (**A**) Volcano plot of differentially expressed genes in HCC when compared with normal tissue. Red nodes represent the significantly up-regulated genes. Purple nodes represent the significantly down-regulated genes. (**B**) Up-regulated genes with significant effect on OS in HCC patients. (**C**) PPI network constructed with genes affecting patient OS.

First, we acquired HCC gene expression profiles from TCGA, and through analysis, we identified several differentially expressed lysosome-related genes. Most of the genes that were identified were up-regulated, with only one gene noted as down-regulated (Figure 1A). Subsequently, we evaluated the impact of these genes on HCC survival. Remarkably, we found some genes could promote cancer development and had notable clinical significance (Figure 1B and Supplementary Figure S1) with regard to overall survival (OS) and progression-free survival (PFS). We used the STRING database and Cytoscape software to build a PPI network using DEGs affecting OS, and these differential genes form two groups, NAGPA and PPT1 may be associated with both groups of genes (Figure 1C).

### Development of OS-related risk signature

**Figure 2:**
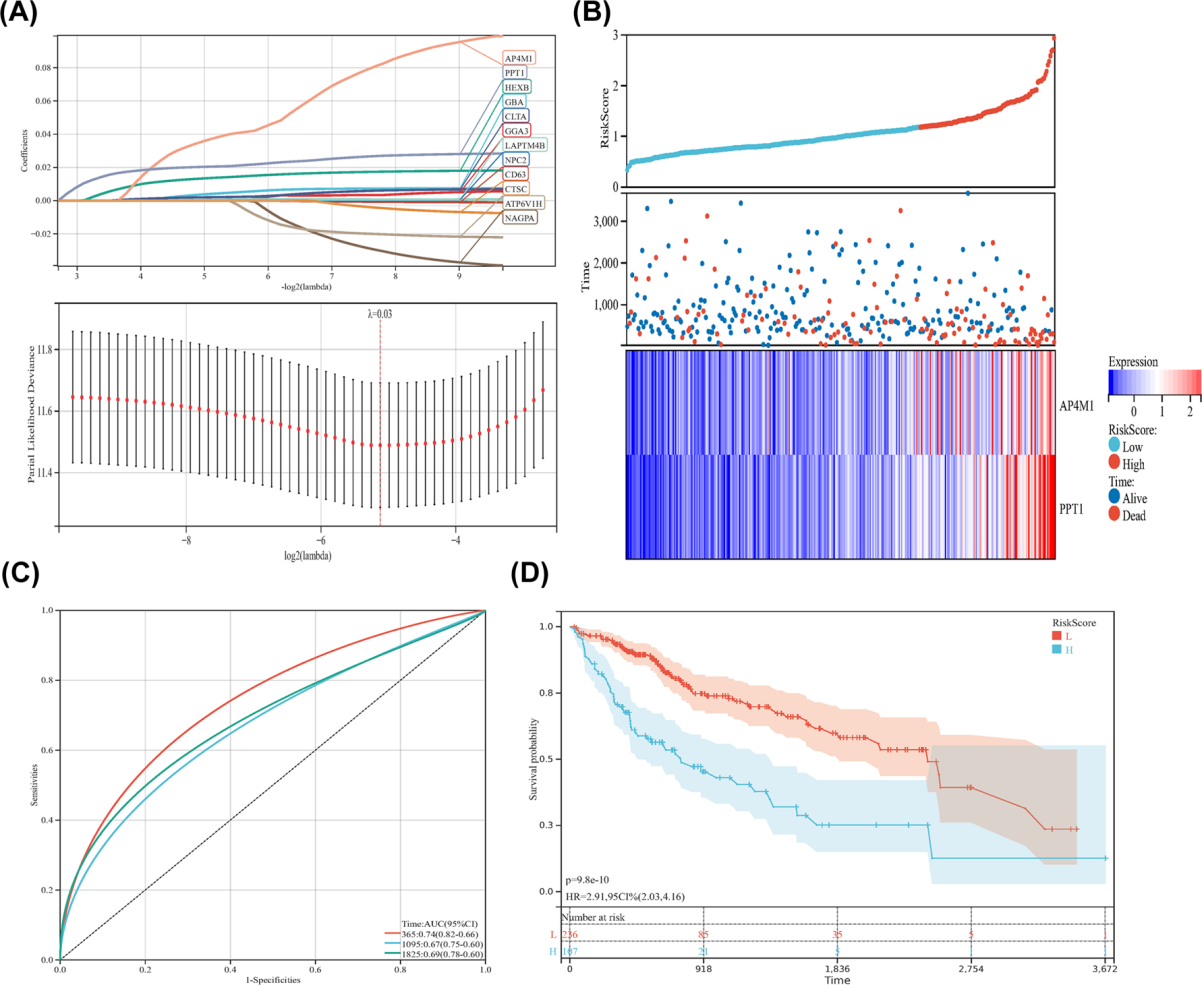
The construction of risk prediction classifier (**A**) Upper, LASSO coefficient profiles of the risk genes. Lower, LASSO regression with 10-fold cross-validation obtained 12 risk genes using minimum lambda value. (**B**) Upper, the curve of risk score. Middle, survival status of the patients. More patients who pass away are correlated with higher risk scores. Lower, heatmap of the expression profiles of AP4M1 and PPT1 in low- and high-risk group. (**C**) Time-dependent ROC analysis of the risk genes. (**D**) Kaplan–Meier survival analysis of the patients in high- and low-risk groups.

LASSO analysis identified 12 genes (AP4MI, PPTI, HEXB, GBA, CLTA, GGA3, LAPTM4B, NPC2, CD63, CTSC, ATP6VIH, and NAGPA), which were included in the classifier (Figure 2A). Based on our calculated risk score, samples were divided into two groups by the median risk score (Figure 2B). In contrast, the high-risk group had a higher mortality rate than the low-risk group (Figure 2B). Two genes (AP4M1, PPT1) were more significantly expressed in the high-risk group than in the low-risk group (Figure 2B). We generated ROC curves, the AUC of the ROC curve predicted survival values for the first year, third year, and fifth year of 0.74, 0.67, and 0.69, respectively, suggesting moderate effectiveness for the prognostic risk model for monitoring survival (Figure 2C). Beyond that, according to Kaplan–Meier survival analysis, OS was significantly lower in the high-risk group than in the low-risk group (Figure 2D).

### Enrichment analysis between high- and low-risk group

**Figure 3:**
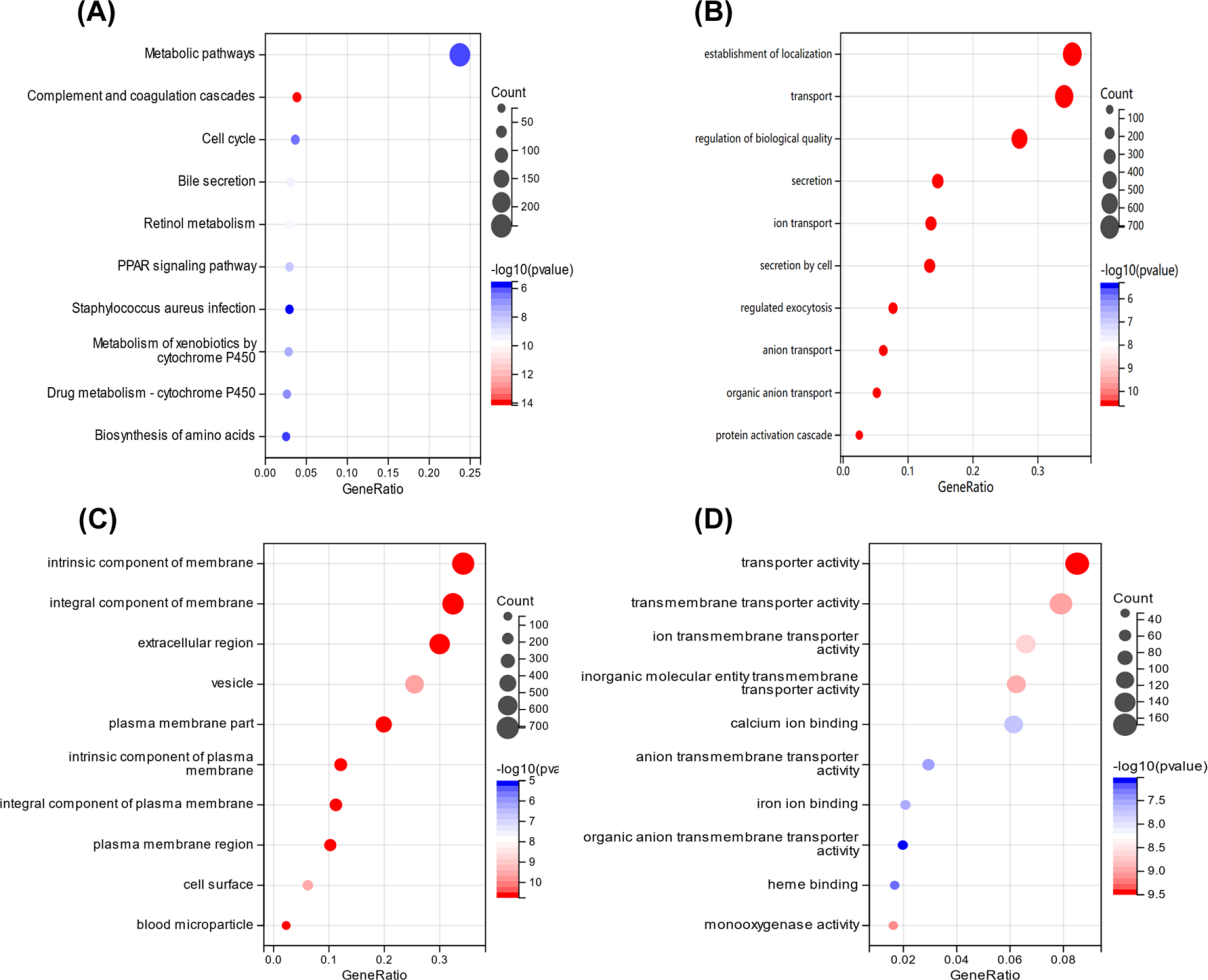
Enrichment of DEGs between high- and low-risk groups (**A**) KEGG enrichment analysis of the DEGs. (**B–D**) Enrichment analysis of GO biological process, cellular component, and molecular function.

Further differential genetic analysis was carried out between high-risk and low-risk samples. A series of DEGs have been obtained (Supplementary Figure S2). To verify biological functions and pathways, the obtained DEGs were analyzed by KEGG pathway analysis and GO enrichment analysis (Figure 3A–D). DEGs were enriched in the KEGG pathway including metabolic pathways, cell cycle and so on (Figure 3A). The DEGs were also obviously enriched in the establishment of localization, transporter activity, and regulation of biological quality (Figure 3B–D). AP4M1and PPT1 may affect downstream related molecules involved in the molecular processes of metabolic and transport functions.

### PPT1 may affect tumor-infiltrating immune cells

**Figure 4:**
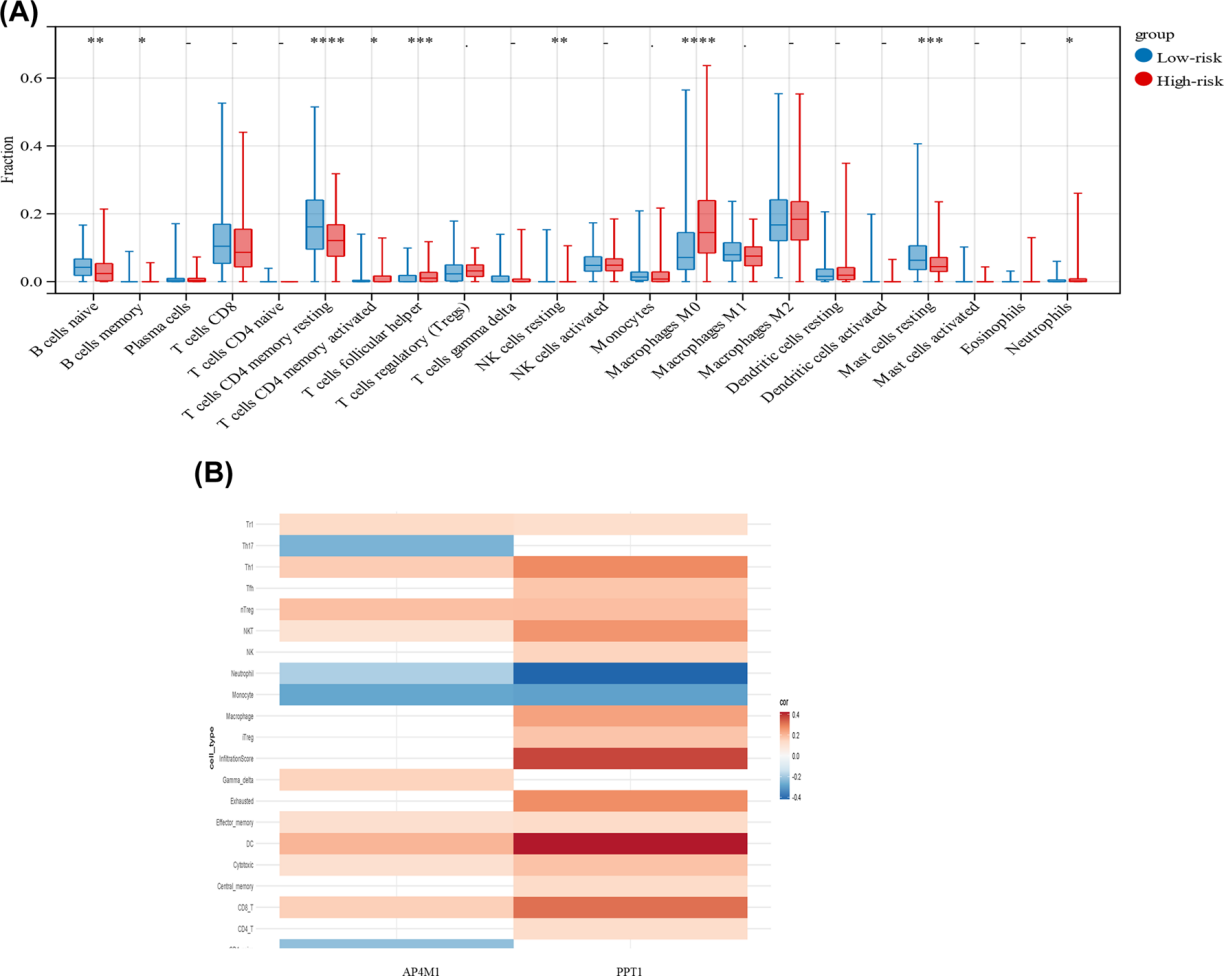
Tumor-immune micro environment analysis of the high- and low-risk groups (**A**) The proportions of immune cells between low- and high-risk samples. The red represents the high-risk group, the blue represents the low-risk group; *P<0.05; **P<0.01; ***P<0.001. (**B**) Correlation heatmap of tumor-infiltrating immune cells and two risk genes.

Tumor cell metabolic activity can have important effects on the tumor microenvironment, leading to local immunosuppression as well as tumor immune escape [18]. The level of immune cell infiltration in the high-risk group and low-risk group was verified. We found that the proportion of macrophages in the high-risk group was higher than that in the low-risk group (Figure 4A). Spearman correlation analysis was used to detect the relationship between genes and tumor-infiltrating immune cells. Compared with AP4M1, macrophages and infiltration score were positively correlated with PPT1 (Figure 4B). Combined with the higher correlation between PPT1 expression and patient survival in Figure 2B, we speculate that PPT1 may play a more dominant role in influencing tumor progression.

### Blockade of PPT1 suppresses the malignant phenotype of HCC cells *in vitro*

**Figure 5:**
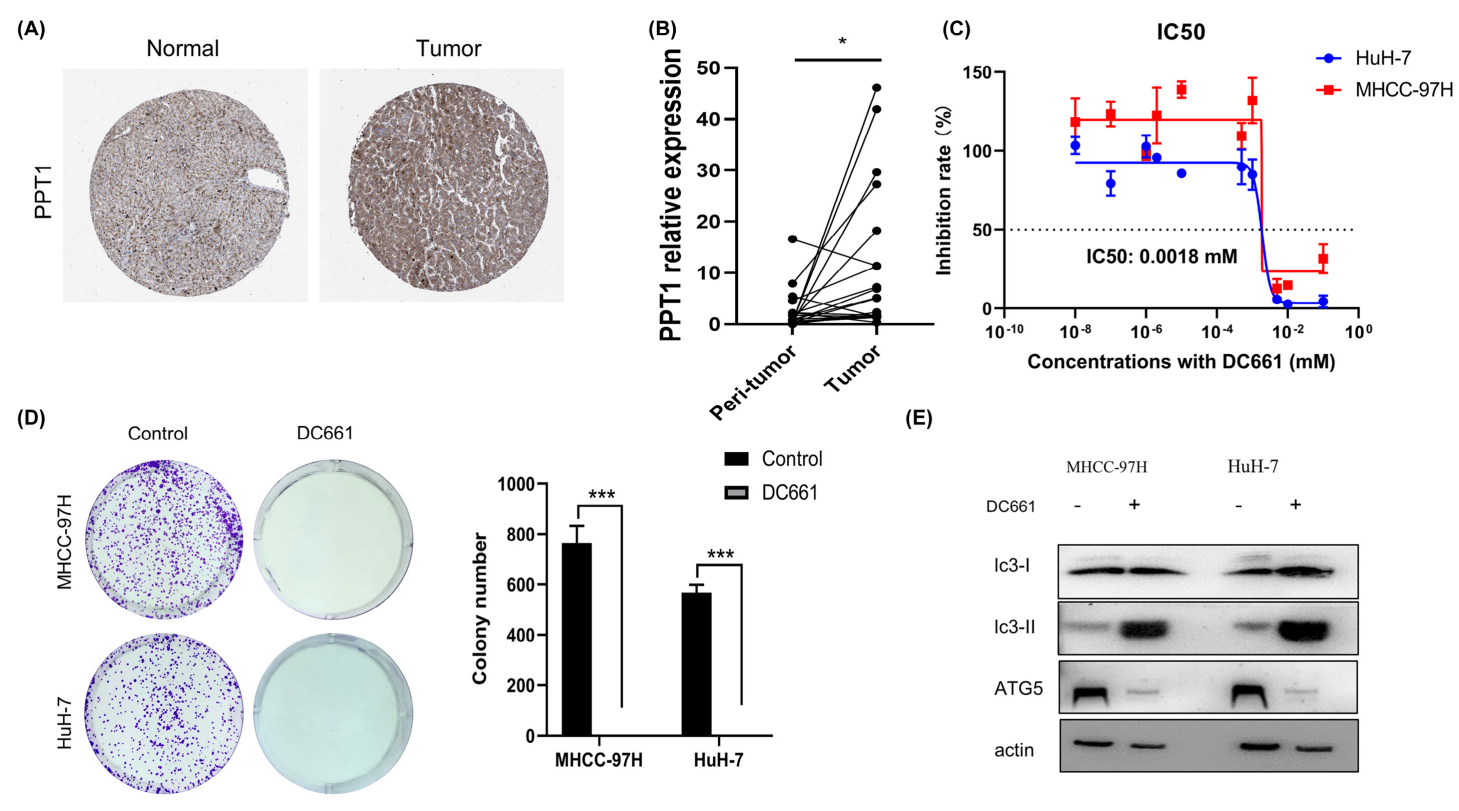
The analysis of the effect of PPT1 on the growth of MHCC-97H and HuH-7 cells (**A**) The protein expression level of PPT1 was higher in HCC tumors than in normal tissues as detected by IHC. The IHC of PPT1 results from the Human Protein Atlas database. (**B**) The mRNA expression of PPT1 was higher in HCC tumors than in paired peritumor samples as detected by qRT-PCR (*n*=20); *P<0.05. (**C**) The CCK8 assay was used to investigate the effect of DC661 on HCC cell viability at different concentrations. (**D**) Representative images and quantification of colony formation assay. HCC cells were chronically treated with DC661 (2 μM, 10 days) for colony formation assays. Cells were subsequently stained with crystal violet and imaged. The number of colonies with >50 cells were scored; ***P<0.001. (**E**) Western blot showing an increase in LC3-II in HCC cells treated with DC661 (2 µM, 24 h).

We analyzed the correlation of PPT1 on HCC at different grades and stages, and with the occurrence of metastasis. The results showed that PPT1 was more highly expressed in higher malignant tumors, expression was positively correlated with tumor metastasis, and PPT1 may play a role in promoting the progression of HCC (Supplementary Figure S3). The protein expression of the genes was determined using immunohistochemistry (IHC) from the Human Protein Atlas database (HPA) to verify the transcriptome analysis results. The protein expression levels of PPT1 showed up-regulated (Figure 5A). The transcription is associated with promoter methylation, TCGA database was used to analyze the expression of PPT1 and its methylation status. We found that the methylation level of PPT1 was negatively correlated with the transcription level (Supplementary Figure S4), and its low methylation status in HCC may have contributed to the up-regulated expression.

In order to validate the bioinformatics analysis results, 20 paired HCC tumor and peri-tumor samples were studied. Real-time PCR was performed on 20 pairs of fresh tumor tissues and adjacent normal liver tissues. Compared with peri-tumor controls, the expression of PPT1 was significantly increased in HCC tissues (Figure 5B), which was consistent with the bioinformatics results obtained by the TCGA dataset.

To investigate why PPT1 affects tumor progression, we analyzed the correlation between PPT1 expression and pathways, and the results showed that high expression of PPT1 in HCC promotes the cell cycle and epithelial–mesenchymal transition (EMT) pathway (Supplementary Figure S5). Therefore, we next investigated the effect of PPT1 on the proliferation of HCC cells through experiments. DC661 is a novel PPT1-targeted inhibitor that exerts an anti-lysosomal function and impairs tumor growth by inhibiting PPT1 [19]. To assess the impact on HCC cells, we carried out the CCK8 assay. Specifically, we found that DC661 had an inhibitory effect on the growth of HCC cell lines MHCC-97H and HuH7 (Figure 5C). Additionally, DC661 (at a concentration of 2 μM) had a marked inhibitory effect on the clonal survival of both cell lines (Figure 5D). Our results confirm that DC661 is capable of significantly inhibiting the growth of HCC cells. To further analyze the effect of PPT1 inhibition on cells, western blot was used to detect the expression of autophagy-related proteins. We observed that treatment with DC661 led to a decrease in the expression level of ATG5. Additionally, DC661 inhibited lysosomes, which resulted in lysosomal dysfunction and restrained the degradation of autophagosomes. This corresponded with the inhibition of late autophagy and the subsequent accumulation of LC3-II (Figure 5E).

### Inhibition of PPT1 affects HCC macromolecular function

Mass spectrometry (MS) is a label-free quantification method that offers more accurate proteome quantification. To explore the potential mechanism that was responsible for the DC661-induced proliferation suppression, quantitative proteomics was used to detect proteomic alterations in the MHCC-97H cells treated with DC661 (2 μM) for 48 h.

**Figure 6:**
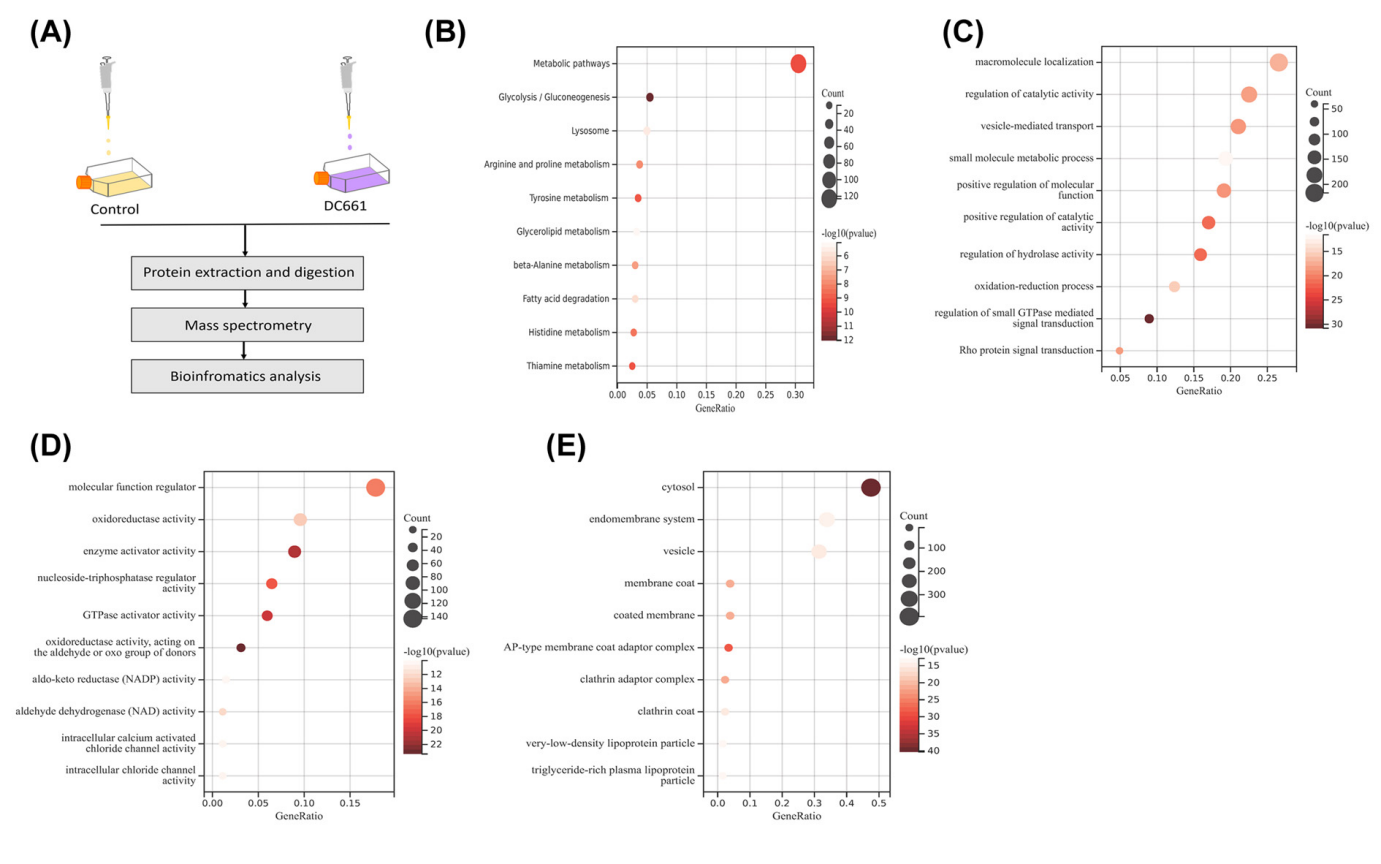
Proteomic analysis and enrichment analysis of differential proteins (**A**) Schematic diagram of LC-MS/MS analysis. (**B**) Enrichment analysis of KEGG pathways was shown. (**C–E**) Enrichment analysis of GO biological process, molecular function, and cellular component.

Total cell proteins were collected, to investigate the overall effect of DC661 on the proteome, global proteomics was analyzed by LC-MS/MS (Figure 6A). Compared to control cells treated with 2 μM of DC661, we observed 280 differential proteins in DC661-treated cells. A total of 99 proteins were expressed up-regulated (Supplementary Table S1) and 181 proteins were expressed down-regulated (Supplementary Table S2). The obtained proteins were analyzed by KEGG pathway analysis and GO enrichment analysis (Figures 6B–E). The proteins were enriched in the KEGG pathway including metabolic pathways, glycolysis/gluconeogenesis, and so on (Figure 6B). The proteins were also obviously enriched in macromolecule localization, regulation of catalytic activity, and vesicle-mediated transport (Figure 6C). Based on proteomics, it is hypothesized that PPT1 may influence the metabolism and function of macromolecules to promote HCC progression.

## Discussion

In the present study, differential lysosome-related genes were screened to construct a prognostic model based on TCGA data, and PPT1, a core lysosome-related gene was found to be significantly upregulated in HCC, which is detrimental to the prognosis of HCC. And the analysis demonstrated that PPT1 as a lysosome-related gene was associated with cell proliferation, autophagy, and immune cell infiltration. Proteomics analysis has shown that PPT1 affects the metabolism, localization, and function of a variety of macromolecular proteins.

The endosomal–lysosomal pathway (ELP) processes proteins through multiple membrane-bound cellular compartments, the proteins endocytosed into the cell are subsequently degraded through early endosomes, endosomal carrier vesicles, late endosomes, and lysosomes [20,21]. The lysosomal pathway usually includes two mechanisms for degrading target proteins [20,22,23], the ELP and the autophagy–lysosomal pathway. ELP is mainly responsible for the degradation of extracellular and transmembrane proteins and plays an important role in nutrient uptake, signaling transduction, antigen presentation, and storage in cells [21]. The autophagy–lysosome phagocytizes intracellular substances, including proteins and other biomolecules [24,25]. These substances are degraded by hydrolases within the lysosomes to generate amino acids, fatty acids, and other substances, which are reused by cells to enable cellular metabolism and energy renewal [26]. Lysosomal helps cancer cells obtain substances necessary for survival to adapt to the stressful environment [27,28], maintain tumor metabolism, growth and survival and carry out subsequent proliferation, migration, and invasion [6], and even eventually mediate tumor resistance to therapeutic drugs [29]. Inhibition of lysosomes has been shown to be a valuable therapeutic tool that can improve the efficacy of cancer treatment by being used in combination with conventional anti-cancer therapies. At present, a variety of small molecule compounds have been developed to kill tumor cells by inducing lysosomal membrane permeability (LMP) or regulating lysosomal function. For example, chloroquine induces LMP to modulate lysosomal function [30], thereby restoring the sensitivity of refractory non-small cell lung cancer cells to cisplatin; salinomycin effectively isolates iron-induced LMP [31], thereby effectively killing tumor cells. LMP has been found to be an effective way to kill many different types of cancer cells, including breast cancer [32,33], ovarian cancer [32], cervical cancer [32], colon cancer [34,35], prostate cancer [32], lung cancer [34], bone cancer [32], skin cancer [34], and AML [36].

Lysosomal effects on tumor progression have been demonstrated, but systematic assessment of which genes are key genes affecting lysosomal function has not been adequately reported in HCC, systematic analysis of which lysosome-related gene playing a dominant role is essential for finding prognostic biomarker and therapeutic target. The function and role of selected genes in the lysosome have been investigated, previous studies have found that PPT1 in tumors correlates with poor survival in patients in a variety of cancers [19]. GNS561, an autophagy inhibitor whose anti-cancer activity is associated with effects on lysosomes, showed potent anti-tumor activity against HCC [37]. PPT1 inhibitor DC661 inhibited autophagy and enhances sorafenib sensitivity in HCC [38]. Inhibition of PPT1 also enhanced anti-PD-1 antibody anti-tumor activity, associated with mediated secretion of IFN-β by macrophages [39], which is somewhat similar to our results analyzing immune cell infiltration. Compared to numerous other lysosomal genes, although these genes show up-regulated expression in HCC, our screen revealed that PPT1 is most associated with the prognosis of HCC. PPT1 may be the most critical of the lysosomal-related genes affecting the progression of HCC.

As summarized in our work by bioinformatics analysis and in vitro experiment, we provided evidence that PPT1 is abnormally expressed in HCC and can be used to predict the prognosis of HCC. PPT1 has an impact on HCC progression, PPT1 deserves more exploration and demonstration for its potentiality in therapeutic targets and molecular mechanisms.

## Supplementary Material

Supplementary Figures S1-S5 and Tables S1-S2

## Data Availability

Publicly available datasets were analyzed in this study, these can be found in The Cancer Genome Atlas (https://portal.gdc.cancer.gov/).

## Abbreviations

DMEM: Dulbecco’s Modified Eagle Medium
ELP: endosomal–lysosomal pathway
EMP: epithelial–mesenchymal transition
HCC: Hepatocellular carcinoma
HPA: Human Protein Atlas database
IHC: Immunohistochemistry
LMP: Lysosomal membrane permeability
PPT1: Palmitoyl protein thioesterase 1
