# Supplementary material for: Identification of PPT1 as a lysosomal core gene with prognostic value in hepatocellular carcinoma
Source: Biosci Rep. 2023 May 18;43(5):BSR20230067. doi: 10.1042/BSR20230067 (PMC10196150; doi:10.1042/BSR20230067)
Supplement: Supplementary Figures S1-S5 and Tables S1-S2 [file BSR-2023-0067_supp.pdf]

# *Supplementary Material*

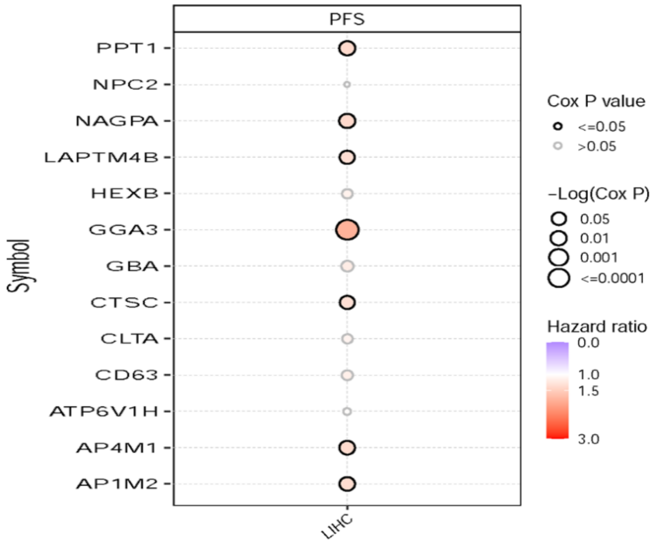


Supplementary FIGURE S1| Effect of upregulated genes on PFS of HCC patients.

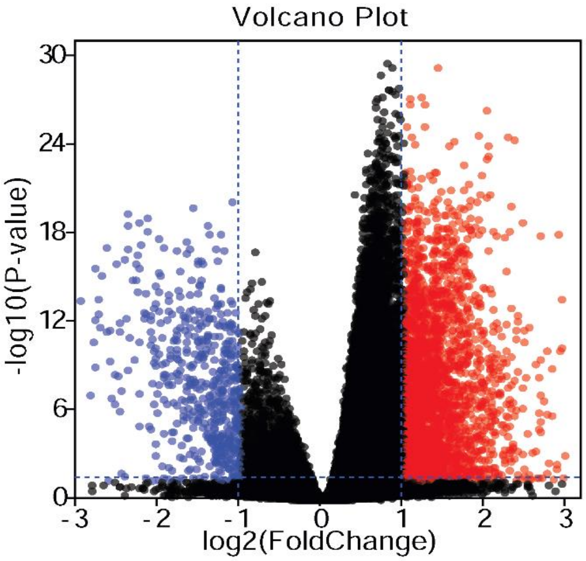


Supplementary FIGURE S2| Volcano plot of DEGs between high and low risk groups.

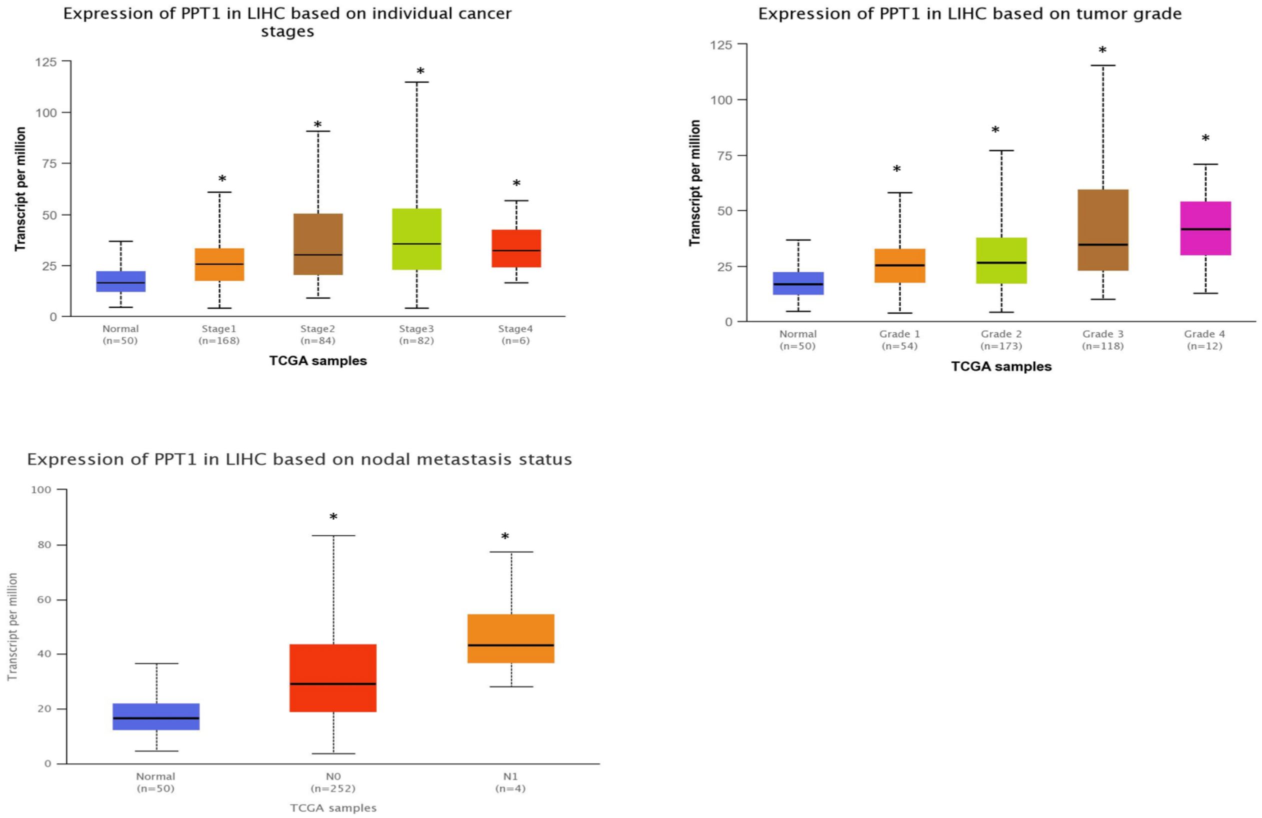


Supplementary FIGURE S3| Correlation between PPT1 expression and the pathology of HCC patients.

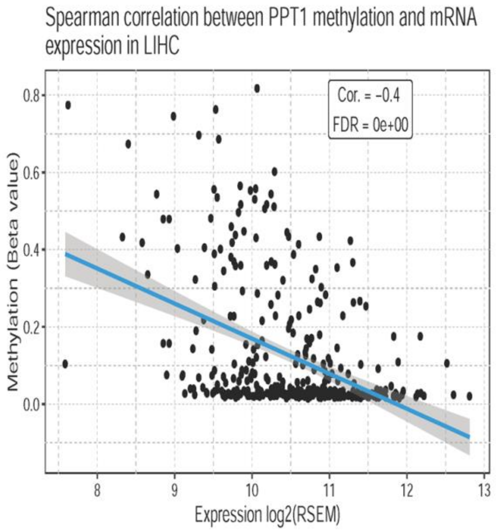


Supplementary FIGURE S4| The relationship between PPT1 methylation and expression in TCGA HCC dataset.

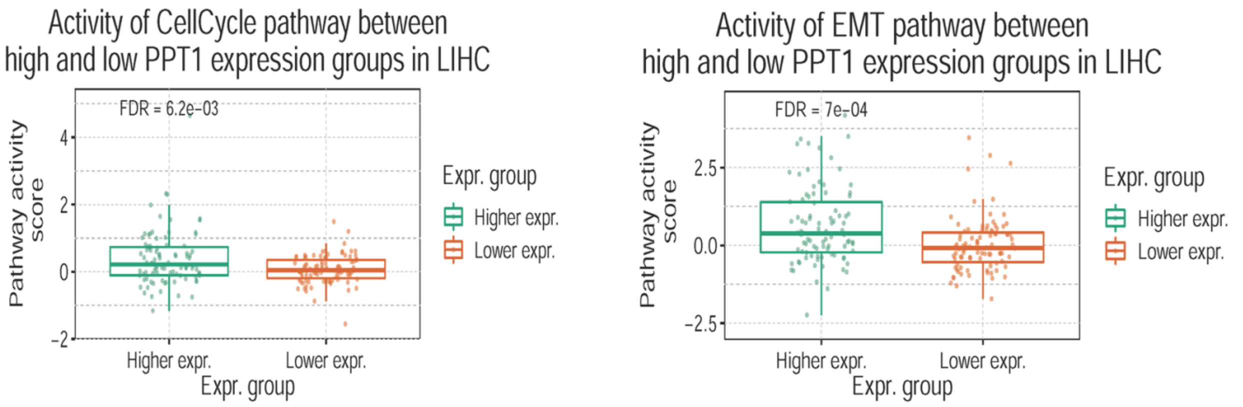


Supplementary FIGURE S5| The correlation between PPT1 expression and cell cycle and Epithelial-Mesenchymal Transitionpathway

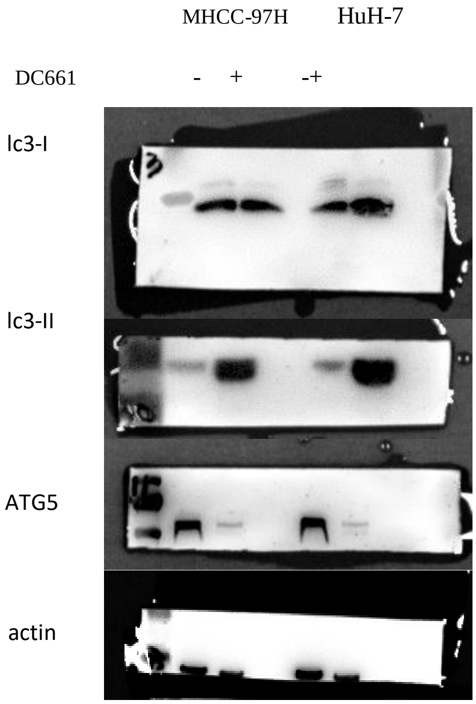


Supplementary FIGURE S6| The full uncropped and unedited versions of Western blots

Supplementary TABLE 1 | List of 99 up-regulated differential proteins in DC661-treated cells

| Name | Expression |
|---|---|
| TUBB2B | up-regulated |
| ATP5F1A | up-regulated |
| DIAPH1 | up-regulated |
| RGPD1 | up-regulated |
| P4HA1 | up-regulated |
| ZFR | up-regulated |
| PLOD3 | up-regulated |
| EIF4A2 | up-regulated |
| EMC1 | up-regulated |
| RBM28 | up-regulated |
| PTCD3 | up-regulated |
| DNCL1 | up-regulated |
| PSMA4 | up-regulated |
| AQR | up-regulated |
| RPL26 | up-regulated |
| FANCI | up-regulated |
| THBS1 | up-regulated |
| PAF1 | up-regulated |

| | |
|---|---|
| ACT | up-regulated |
| NDC1 | up-regulated |
| GPX8 | up-regulated |
| ABCC1 | up-regulated |
| HMGN1 | up-regulated |
| WDR4 | up-regulated |
| SMARCB1 | up-regulated |
| KIF2A | up-regulated |
| DKFZp686P18130 | up-regulated |
| TMEM209 | up-regulated |
| C9orf64 | up-regulated |
| ATXN2 | up-regulated |
| GATAD2A | up-regulated |
| KDSR | up-regulated |
| PTP4A1 | up-regulated |
| LARP7 | up-regulated |
| CEP170 | up-regulated |
| GTF3C2 | up-regulated |
| DPM1 | up-regulated |
| ABCB10 | up-regulated |

| | |
|---|---|
| AIFM2 | up-regulated |
| ARF6 | up-regulated |
| UBE3C | up-regulated |
| SARS2 | up-regulated |
| TFB2M | up-regulated |
| SCO2 | up-regulated |
| MRPL22 | up-regulated |
| C6orf11 | up-regulated |
| HMGN2 | up-regulated |
| TRRAP | up-regulated |
| ATPAF1 | up-regulated |
| SEC24A | up-regulated |
| VTN | up-regulated |
| PPHLN1 | up-regulated |
| YLPM1 | up-regulated |
| CAT | up-regulated |
| GSDME | up-regulated |
| FAM136A | up-regulated |
| NEDD4 | up-regulated |
| ZFP36L2 | up-regulated |
| UAP1L1 | up-regulated |

| | |
|---|---|
| SYNE1 | up-regulated |
| MTRR | up-regulated |
| NCAPD3 | up-regulated |
| ACAP2 | up-regulated |
| TDP2 | up-regulated |
| CNN2 | up-regulated |
| TBC1D5 | up-regulated |
| PODXL | up-regulated |
| COG5 | up-regulated |
| ELOVL1 | up-regulated |
| COL1A2 | up-regulated |
| DOCK7 | up-regulated |
| OSBPL9 | up-regulated |
| COG6 | up-regulated |
| PEX1 | up-regulated |
| WASHC5 | up-regulated |
| AURKAIP1 | up-regulated |
| RAB19 | up-regulated |
| CHMP5 | up-regulated |
| PREB | up-regulated |

| | |
|---|---|
| WTAP | up-regulated |
| SUZ12 | up-regulated |
| MOSPD2 | up-regulated |
| MRPL10 | up-regulated |
| MED22 | up-regulated |
| FAM91A1 | up-regulated |
| TIMP3 | up-regulated |
| PAFAH2 | up-regulated |
| SAP30 | up-regulated |
| SOGA | up-regulated |
| DKFZp686N1815 | up-regulated |
| MAPK8IP1 | up-regulated |
| TOPAZ1 | up-regulated |
| ABCD2 | up-regulated |
| RP2 | up-regulated |
| CCDC93 | up-regulated |
| RAB23 | up-regulated |
| ZNF560 | up-regulated |
| DOK3 | up-regulated |
| PSME4 | up-regulated |

Supplementary TABLE 2 | List of 181 down-regulated differential proteins in DC661-treated cells

| Name | Expression |
|---|---|
| FLNB | down-regulated |
| TUBB4A | down-regulated |
| TPR | down-regulated |
| TUBB3 | down-regulated |
| TKT | down-regulated |
| TRAP1 | down-regulated |
| LIMA1 | down-regulated |
| ALPG | down-regulated |
| SF3A1 | down-regulated |
| SET | down-regulated |
| HEL2 | down-regulated |
| EEF1D | down-regulated |
| HMGB2 | down-regulated |
| HNRNPH2 | down-regulated |
| SSB | down-regulated |
| DPYSL2 | down-regulated |

| | |
|---|---|
| FKBP4 | down-regulated |
| RBBP7 | down-regulated |
| PGAM1 | down-regulated |
| NME1 | down-regulated |
| DBN1 | down-regulated |
| EMD | down-regulated |
| TUBG1 | down-regulated |
| NAMPT | down-regulated |
| TRIP6 | down-regulated |
| MTCH2 | down-regulated |
| RCN1 | down-regulated |
| KLC2 | down-regulated |
| KRT10 | down-regulated |
| PSMD6 | down-regulated |
| EIF3S1 | down-regulated |
| EIF5A | down-regulated |
| P4HA2 | down-regulated |
| HINT1 | down-regulated |
| DNAJB11 | down-regulated |
| KRT2 | down-regulated |
| YWHAH | down-regulated |

| | |
|---|---|
| CRYZ | down-regulated |
| CKAP5 | down-regulated |
| RPAP3 | down-regulated |
| SNRPD2 | down-regulated |
| U2AF1 | down-regulated |
| ABHD10 | down-regulated |
| GPKOW | down-regulated |
| SEPTIN2 | down-regulated |
| ALDH1B1 | down-regulated |
| NUP37 | down-regulated |
| HNRNPLL | down-regulated |
| ARHGEF1 | down-regulated |
| PHPT1 | down-regulated |
| LAP3 | down-regulated |
| CACYBP | down-regulated |
| GCLM | down-regulated |
| PPP1R12A | down-regulated |
| POLD2 | down-regulated |
| EDC4 | down-regulated |
| ZYX | down-regulated |

| | |
|---|---|
| BTF3 | down-regulated |
| GOLGA2 | down-regulated |
| GDI1 | down-regulated |
| CSNK2A2 | down-regulated |
| BRD4 | down-regulated |
| NRAS | down-regulated |
| UCHL3 | down-regulated |
| TACO1 | down-regulated |
| DENR | down-regulated |
| GFAP | down-regulated |
| RCN3 | down-regulated |
| GIPC1 | down-regulated |
| NAA10 | down-regulated |
| IKBIP | down-regulated |
| ETFDH | down-regulated |
| MEPCE | down-regulated |
| WDR74 | down-regulated |
| C12orf10 | down-regulated |
| UCK2 | down-regulated |
| SF3A2 | down-regulated |
| C10orf70 | down-regulated |

| | |
|---|---|
| ADGRE5 | down-regulated |
| MRPL32 | down-regulated |
| RPS15 | down-regulated |
| RTN3 | down-regulated |
| H2AC21 | down-regulated |
| RPL7L1 | down-regulated |
| GRPEL1 | down-regulated |
| STK10 | down-regulated |
| PHF6 | down-regulated |
| TTC9C | down-regulated |
| MCMBP | down-regulated |
| RAB3GAP1 | down-regulated |
| HEL-S-95n | down-regulated |
| LAMTOR1 | down-regulated |
| ANAPC7 | down-regulated |
| TMED4 | down-regulated |
| RRP7A | down-regulated |
| NEDD8 | down-regulated |
| SFN | down-regulated |
| UTP14A | down-regulated |

| | |
|---|---|
| TCEA1 | down-regulated |
| AHSG | down-regulated |
| NVL | down-regulated |
| MICOS13 | down-regulated |
| NSA2 | down-regulated |
| AP1S1 | down-regulated |
| PAIP1 | down-regulated |
| NOL7 | down-regulated |
| EIF2D | down-regulated |
| NDUFAB1 | down-regulated |
| NOL11 | down-regulated |
| LASP1 | down-regulated |
| STXBP1 | down-regulated |
| MMGT1 | down-regulated |
| TREX1 | down-regulated |
| FAM162A | down-regulated |
| NPM3 | down-regulated |
| LARP4 | down-regulated |
| TIPRL | down-regulated |
| XPO7 | down-regulated |
| PRCC | down-regulated |

| | |
|---|---|
| MOB1B | down-regulated |
| SNRPD3 | down-regulated |
| GLT8D1 | down-regulated |
| NDUFB5 | down-regulated |
| PCBD1 | down-regulated |
| FAM49B | down-regulated |
| PBR | down-regulated |
| HTRA2 | down-regulated |
| CDKN2AIP | down-regulated |
| PQBP1 | down-regulated |
| DHX38 | down-regulated |
| GTF3C3 | down-regulated |
| SIGMAR1 | down-regulated |
| PTPMT1 | down-regulated |
| RPL36A | down-regulated |
| NCKAP1 | down-regulated |
| TMED5 | down-regulated |
| SPCS2 | down-regulated |
| DYNC1LI2 | down-regulated |
| HSPC148 | down-regulated |

| | |
|---|---|
| CDK4 | down-regulated |
| SDC4-ROS1_S4;R34 | down-regulated |
| NOP16 | down-regulated |
| FAM50A | down-regulated |
| NDUFB3 | down-regulated |
| QTRT2 | down-regulated |
| GBF1 | down-regulated |
| SCRN1 | down-regulated |
| ZNF346 | down-regulated |
| RPA3 | down-regulated |
| RBX1 | down-regulated |
| ATG3 | down-regulated |
| CARS2 | down-regulated |
| NEMF | down-regulated |
| MARCHF5 | down-regulated |
| TMX3 | down-regulated |
| SF3B5 | down-regulated |
| TIGAR | down-regulated |
| CHAMP1 | down-regulated |
| MAK16 | down-regulated |
| THOC7 | down-regulated |

| | |
|---|---|
| PDF | down-regulated |
| NACAD | down-regulated |
| CRIP1 | down-regulated |
| TRIM56 | down-regulated |
| DCAF8 | down-regulated |
| LSM4 | down-regulated |
| RANBP9 | down-regulated |
| PAPOLA | down-regulated |
| CASP4 | down-regulated |
| PPP4R3A | down-regulated |
| PLCB3 | down-regulated |
| EHBP1L1 | down-regulated |
| PTRH1 | down-regulated |
| VBP1 | down-regulated |
| CHKA | down-regulated |
| VPS25 | down-regulated |
| ICMT | down-regulated |
| TIMM10 | down-regulated |
| NUBP1 | down-regulated |
| CTSC | down-regulated |

| | |
|---|---|
| EPS15 | down-regulated |

Supplementary TABLE 3| List of abbreviations

| Abbreviation | Definition |
|---|---|
| HCC | Hepatocellular Carcinoma |
| TCGA | The Cancer Genome Atlas |
| DEGs | Differentially Expressed Genes |
| PPT1 | Palmitoyl Protein Thioesterase 1 |
| LRGs | Lysosome-related Genes |
| MSigDB | The Molecular Signatures Database |
| PPI | Protein-protein Interaction |
| LASSO | Least Absolute Shrinkage and Seletion Operator |
| RS | Risk Score |
| ROC | Receiver Operating Characteristic Curve |
| AUC | Area Under Curve |
| OS | Overall Survival |
| GO | Gene Ontology |

| | |
|---|---|
| KEGG | Kyoto Encyclopedia of Genes and Genomes |
| DMEM | Dulbecco’s Modified Eagle Medium |
| FBS | Fetal Bovine Serum |
| CCK8 | Cell Counting Kit 8 |
| IHC | Immunohistochemistry |
| HPA | Human Protein Atlas Database |
| MS | Mass spectrometry |
| ELP | Endosomal-lysosomal Pathway |
| ALP | Autophagy-lysosomal Pathway |
| LMP | Lysosomal Membrane Permeability |
